# Linc00152 suppresses apoptosis and promotes migration by sponging miR-4767 in vascular endothelial cells

**DOI:** 10.18632/oncotarget.18777

**Published:** 2017-06-28

**Authors:** Wei Teng, Chunguang Qiu, Zhaohui He, Guoliang Wang, Yongliang Xue, Xuezhi Hui

**Affiliations:** ^1^ Department of Cardiology, The First Affiliated Hospital of Zhengzhou University, Zhengzhou 450052, China; ^2^ Department of Cardiology, The First Affiliated Hospital of Henan University, Kaifeng 475000, China

**Keywords:** vascular endothelial cells, linc00152, cell apoptosis and migration, miR-4767, ceRNA

## Abstract

Dysfunction of vascular endothelial cells (VECs), such as increased apoptosis and diminished migration, is closely connected with most cardiovascular diseases and angiogenesis-related events. LncRNAs have been involved in regulation of many pathological processes, but their roles in vascular endothelial function are hardly underreported. Here, we explore the role of a intergenic lncRNA named linc00152 in the apoptosis and migration of VECs. We found that linc00152 was downregulated in human umbilical vein VECs (HUVECs) in a dose- and time-dependent manner following treatment with oxLDL, which is a typical proinflammatory factor in the initiation and progression of vascular endothelial dysfunction. Gain- and loss-function experiments indicated that linc00152 distinctly inhibited apoptosis and improved migration in oxLDL-treated HUVECs. By sponging miR-4767, linc00152 positively regulated the expression of Bcl2L12 and EGFR proteins. Moreover, blocking miR-4767 rescued the decrease of Bcl2L12 and EGFR caused by linc00152 knockdown, as well as the changes in cell apoptosis and migration. Our findings propose a novel role of linc00152 in the improvement of vascular endothelial function and a potential target for the therapy of some cardiovascular diseases.

## INTRODUCTION

Long noncoding RNAs (lncRNAs) are non-protein coding transcripts with length longer than 200 nucleotides. It has been widely recognized that lncRNAs constitute a large portion (about 4-9%) of mammalian transcriptomes and that the protein coding transcripts constitute only about 1% [1, 2]. Increasing evidence reveals that lncRNAs participate in nearly all aspects of gene expression regulation, including epigenetics, transcription and post-transcription, and are involved in many biological processes under both physiological and pathological conditions, such as cell growth and movement, differentiation and reprogramming, and stress response [3–6]. The molecular mechanisms by which lncRNAs function in biological processes are abundant. They may directly bind with specific DNA strands, RNA strands and protein molecules to affect transcription, splicing or translation, or they may recruit RNAs and proteins in the cytoplasm or nucleus to form a functional complex [7]. Among these mechanisms, competing endogenous RNA (ceRNA) was first uncovered in muscle differentiation, describing the cross talk between lncRNAs and other transcripts which share the same miRNAs [8, 9].

Fine regulation of gene expression, at the transcriptional and post-transcriptional levels, is required in the response of mammalian cells to internal and external stimuli. Transcriptional regulation achieves by alternation in the composition of transcription factor-promoter complex [10], while post-transcriptional regulation is uausally mediated by alternative splicing of RNA transcripts, alterations in the stability and transport of mRNAs [11]. LncRNAs are the most prevalent and functionally diverse regulator in gene fine regulation. They can function at relatively low expression level and their regulation can be triggered by stimuli during various pathological processes including vascular injury and remodeling [12–14]. Vascular endothelial cells (VECs) lie in the innermost of blood vessels. They are the most abundant component of blood vessel, and they are vulnerable to stress. Dysfunction of VECs is important in pathogenesis of many cardiovascular diseases, such as arteriosclerosis, arteritis, thrombus and plaque erosion [15–19]. Protein-coding genes involved in the regulaion of VEC functions have been relatively well studied. The role of lncRNAs in regulating atherosclerosis and other cardiovascular diseases has attracted interest [20], but the exact role of lncRNAs in VEC functions was largely unknown.

Recently, it was revealed that a famous lncRNA, long intergenic non-coding RNA (lincRNA) p21, promotes cell apoptosis and cell-cycle progression in many types of cells including VECs [21]. Several other studies have shown that lncRNAs could regulate autophagy, inflammation and stress-induced cell death in VECs. For example, lncRNA LOC100129973, partially overlaps with guanine nucleotide-binding protein subunit beta-5 (GNB5) could suppress Bcl-2-mediated apoptosis of VECs by targeting miR-4707-5p and miR-4767 [22]. Another example is that lncRNA TGFB2 overlapping transcript 1 (TGFB2-OT1), transcribed from the antisense strand of the transforming growth factor beta 2 (TGFB2) gene,can bind to miR-3960/-4488/-4459 and promote their target genes mediated autophagy and inflammation of vascular endothelial cells [23]. The migration of VECs plays key roles in angiogenesis, endothelial injury healing and tissue wound healing. Few reports have investigated the exact role of lncRNAs in VEC migration. In this study, we explore the role of lincRNA-00152 (linc00152) in the VEC dysfunction induced by oxidized low density lipoprotein (ox-LDL). We found that linc00152 was downregulated by ox-LDL in a dose- and time-dependent manner and that it improved cell survival and migration.

## RESULTS

### Long intergenic non-coding RNA linc00152 was downregulated by oxLDL treatment in HUVECs

**Figure 1:**
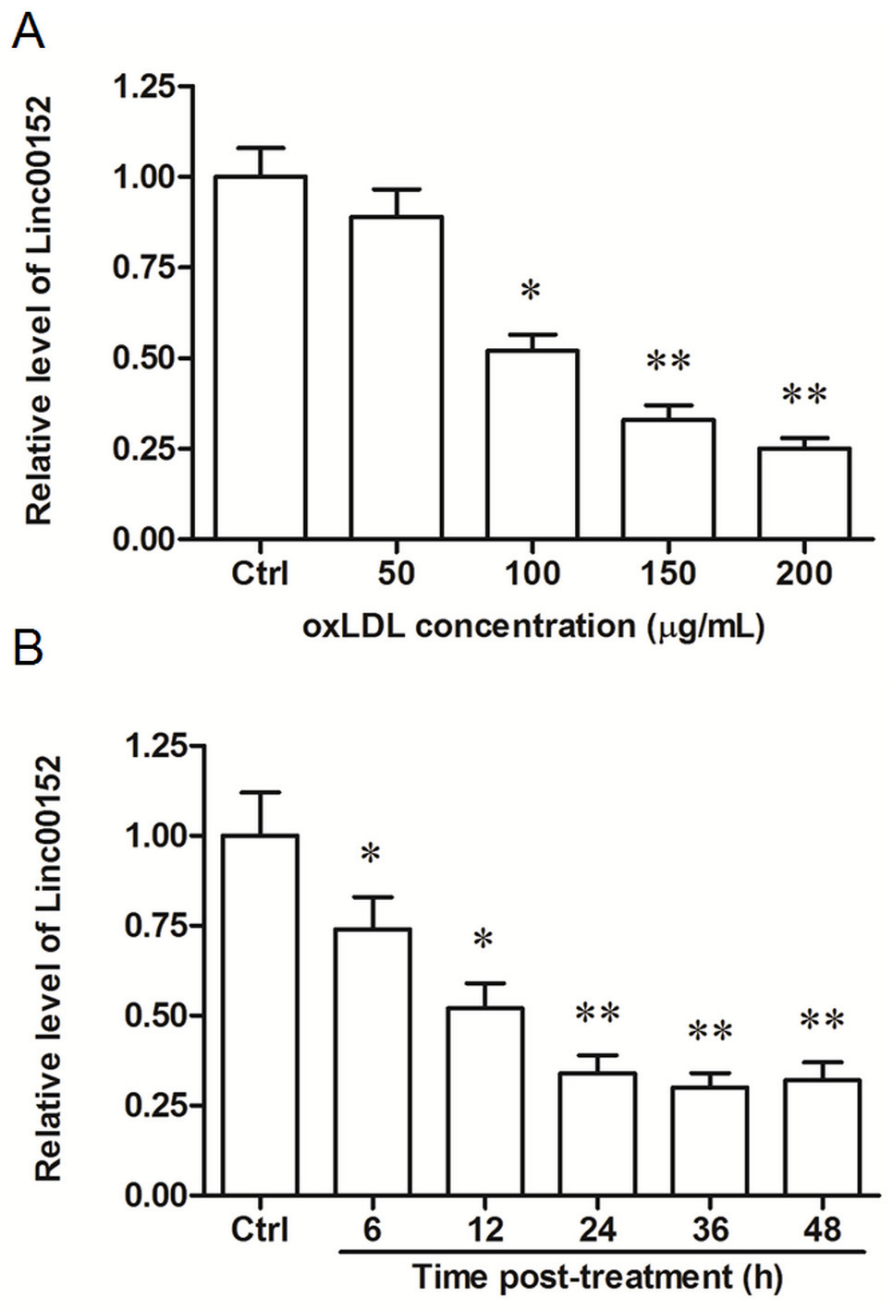
Linc00152 was downregulated by oxLDL treatment in HUVECs in a dose- and time-dependent manner **(A)** Linc00152 was downregulated by oxLDL treatment in HUVECs in a dose-dependent manner. HUVECs were subcultured in 12-well culture plates. On reaching 50% confluence, ox-LDL was added into the cells at final concentrations of 50, 100, 150 and 200 μg/mL. As a control, the cells were incubated with an equal volume of PBS. After incubation for 24 h, the expression of linc00152 was detected by qPCR. **(B)** Linc00152 was downregulated by oxLDL treatment in HUVECs in a time-dependent manner. HUVECs were incubated with 150 μg/mL ox-LDL. At the time points 6 h, 12 h, 24 h, 36 h and 48 h, the expression of linc00152 was detected by qPCR. *P < 0.05, **P < 0.01.

We examined the expression pattern of linc00152 (Genbank accession number: NR_024204.1) in HUVECs following treatment of oxLDL, a comfirmed cause of vascular endothelial cell injury and atherosclerosis. The qPCR results showed that linc00152 was downregulated by oxLDL treatment in a dose- and time-dependent manner (Figure 1A and 1B): after incubating for 24 h, 100 μg/mL oxLDL reduced the linc00152 level by about 50% (P < 0.05), and 150 or 200 μg/mL oxLDL further decreased the linc00152 expression (both by about 70%, P < 0.01); when incubated with 150 μg/mL oxLDL, the linc00152 level was significantly downregulated at 6 h and 12 h (P < 0.05), and its level was further decreased at 24, 36 and 48 h (all by about 70%, P < 0.01).

### Linc00152 suppressed oxLDL-induced apoptosis and promoted migration in HUVECs

**Figure 2:**
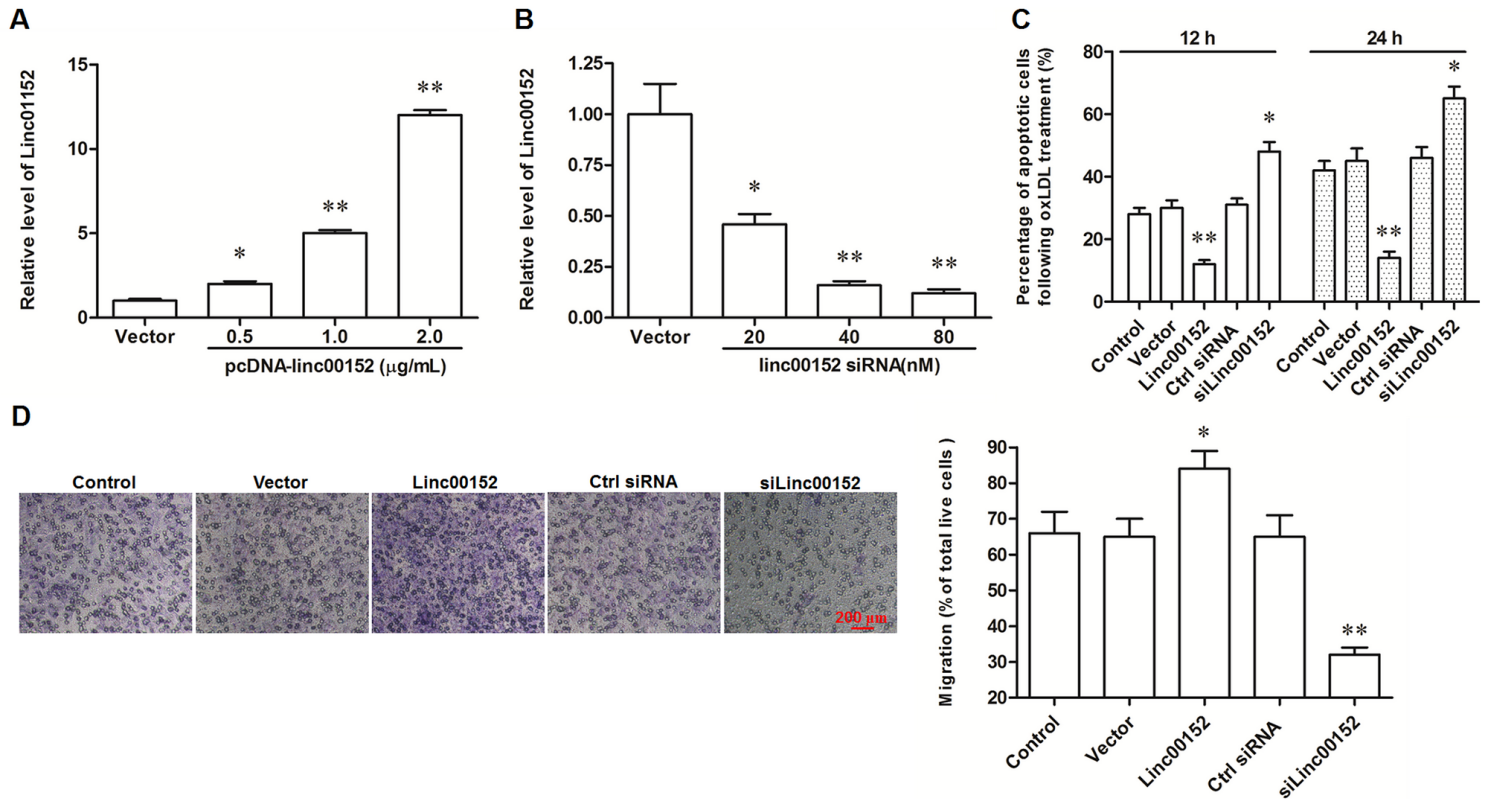
Linc00152 suppressed apoptosis and promoted migration in ox-LDL treated HUVECs Linc00152 overexpression **(A)** and knock down **(B)** efficiencies detected by qPCR analysis. HUVECs were transfected with pcDNA3.1-linc00152 at concentrations of 0.5, 1.0, and 2.0 μg/mL or Linc00152 siRNA at 20, 40, and 80 nM for 48 h. Then, the cells were treated with 150 μg/mL ox-LDL for 24 h and the expression of linc00152 was detected by qPCR. Linc00152 negatively regulated apoptosis **(C)** and positively regulated migration **(D)** in ox-LDL treated HUVECs. HUVECs were transfected with 1.0 μg/mL pcDNA3.1-linc00152 or 40 nM Linc00152 siRNA for 48 h. Then, the cells were treated with 150 μg/mL ox-LDL for 24 h. Cell apoptosis was checked by TUNEL assay and cell migration was detected with the Transwell Migration assay. *P < 0.05, **P < 0.01 *vs.* Vector or Ctrl siRNA.

To investigate the exact role of linc00152 in VECs, the full-length linc00152 was cloned into the pcDNA3.1 expression vector (pcDNA3.1-linc00152), and specific siRNAs against linc00152 (siLinc00152) were designed and synthesized. HUVECs were transfected with pcDNA3.1-linc00152 (at concentrations of 0.5, 1 or 2 μg/mL) or siLinc00152 (20, 40, or 80 nM). The efficiency of overexpression and knockdown were confirmed by qPCR (Figure 2A and 2B). Then, HUVECs were transfected with 1 μg/mL pcDNA3.1-linc00152 or 40 nM siLinc00152. After treatment with 150 μg/mL oxLDL or nothing, cell apoptosis and migration were respectively detected. Linc00152 overexpression caused a modest decrease in cell apoptosis in HUVECs without oxLDL treatment (Supplementary Figure 1), while distinctly suppressed cell apoptosis at 12 h and 24 h (Figure 2C). Whereas linc00152 knockdown dramatically increased cell apoptosis in oxLDL treated and untreated HUVECs (Figure 2C and Supplementary Figure 1). In addition, linc00152 overexpression distinctly increased the migration capacity of HUVECs, and linc00152 knockdown reduced their migration capacity (Figure 2D).

### Linc00152 functioned as an endogenous sponge of miR-4767

**Figure 3:**
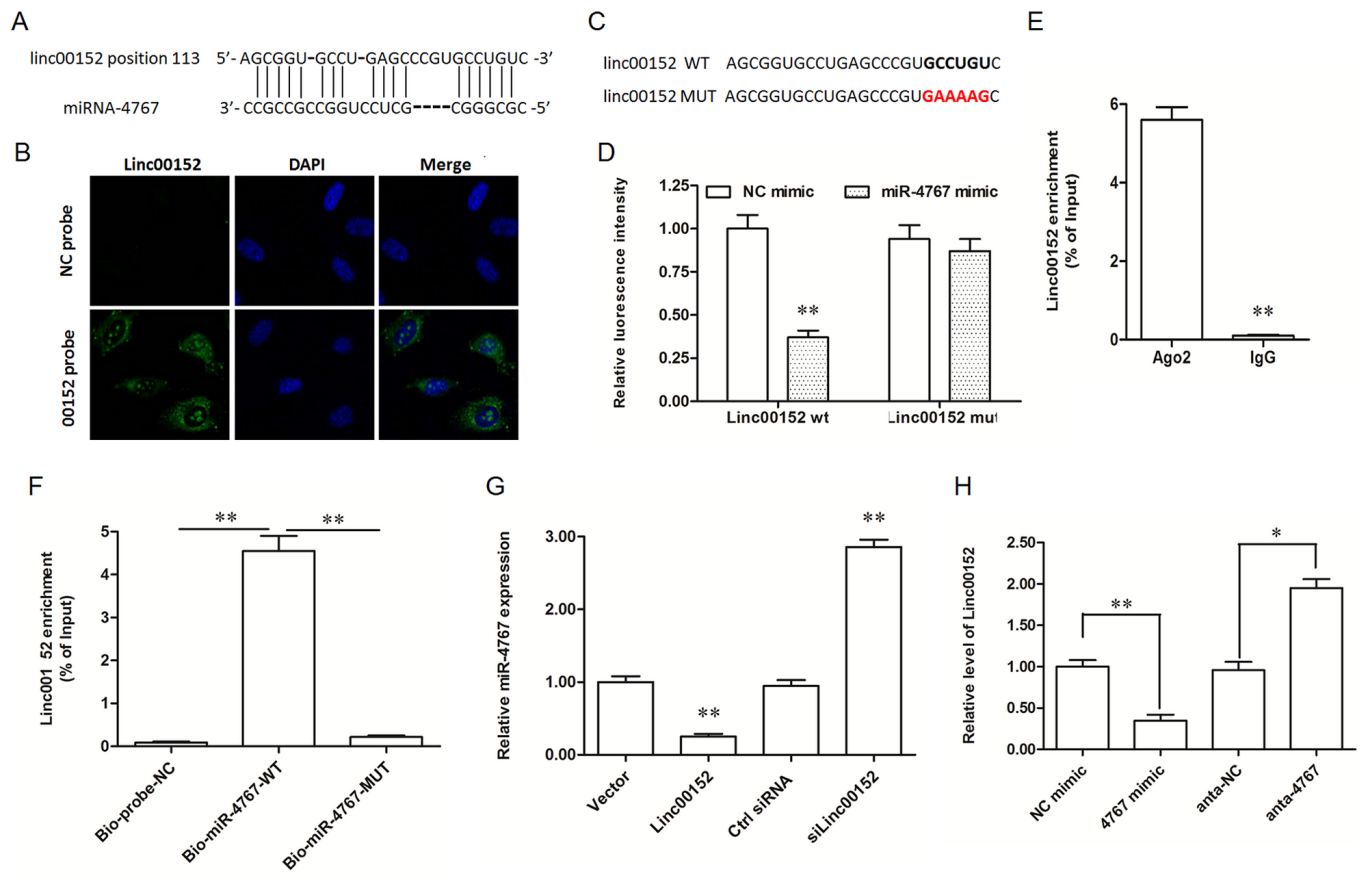
Linc00152 directly bound with miR-4767 and decreased miR-4767 expression level **(A)** Potential site targeted by miR-4767 in linc00152 sequence. **(B)** Linc00152 location in HUVECs detected by RNA fluorescent *in situ* hybridization. The antisense probe was used as a negative control. The images were captured at ×400 amplification. **(C)** Wild type (wt) and mutated (mut) sequences of linc00152 inserted into the luciferase reporter gene vectors. The bold colorful letters indicate the detail of mutated sequences. **(D)** Luc-linc00152-wt or Luc-linc00152-mut vectors were co-transfected into HEK293T cells with 40 nM miR-4767 mimic or control mimic for 48 h and luciferase activity was measured. **(E)** RNA-IP experiments were performed using Ago2 antibody in HUVECs. IgG was used as a negative control. **(F)** RNA pull-down assay with biotinylated miR-4767-wt and miR-4767-mut probes in HUVECs. A scrambled biotinylated miRNA was used as the negative control (NC). **(G)** The effect of overexpression or knockdown of linc00152 on the expression of miR-4767 in HUVECs. HUVECs were transfected with 1.0 μg/mL pcDNA3.1-linc00152 or 40 nM linc00152 siRNA for 48 h. The expression of miR-4767 was detected with qPCR. **(H)** The effect of overexpression or knockdown of miR-4767 on the expression of linc00152 in HUVECs. HUVECs were transfected with 40 nM miR-4767 mimics or 20 nM miR-4767 antagomirs for 48 h. The expression of linc00152 was detected with qPCR. *P < 0.05, **P < 0.01 *vs.* respective controls.

LncRNAs may act as competing endogenous RNAs (ceRNAs) that interact with specific miRNAs and influence miRNA-mediated biological processes. To explore the underlying mechanism through which linc00152 affected the apoptosis and migration of HUVECs, we searched the miRNAs that bind with linc00152 by Micro Inspector (http://bioinfo.uni-plovdiv.bg/microinspector/) and RNAhybrid (http://bibiserv.techfak.uni-bielefeld.de/rnahybrid/). The results showed that there is a binding site for miR-4767 (Figure 3A) and that the binding free energy is very low (-32.6 kcal/mol, Supplementary Figure 2). To validate the prediction, a wide type (WT) and a mutated (MUT) linc00152 luciferase reporter gene vector were constructed (Figure 3B).

**Figure 4:**
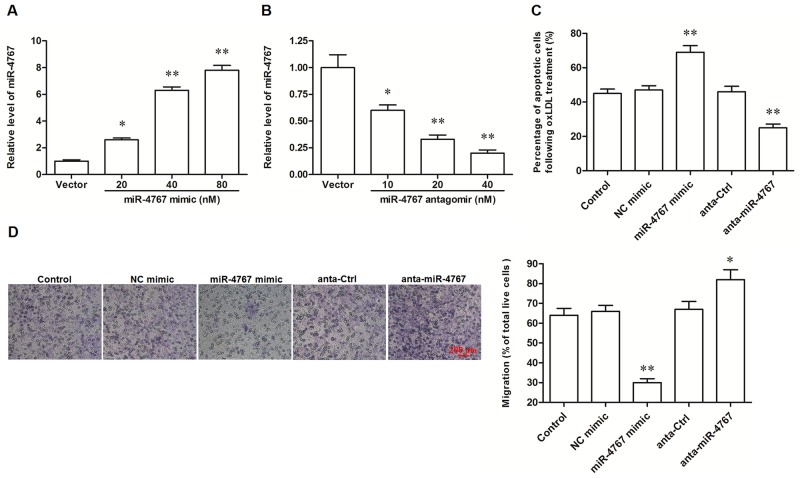
MiR-4767 increased apoptosis and attenuated migration in ox-LDL treated HUVECs MiR-4767 overexpression **(A)** and knock down **(B)** efficiencies detected by qPCR analysis. HUVECs were transfected with miR-4767 mimics at concentrations of 20, 40, and 80 nM or miR-4767 antagomirs at 10, 20, and 40 nM for 48 h. Then, the cells were treated with 150 μg/mL ox-LDL for 24 h and the expression of miR-4767 was detected by qPCR. MiR-4767 increased apoptosis **(C)** and attenuated migration **(D)** in HUVECs. HUVECs were transfected with 20 nM miR-4767 mimics or 40 nM miR-4767 antagomirs for 48 h. Then, the cells were treated with 150 μg/mL ox-LDL for 24 h. Cell apoptosis was checked by TUNEL assay and cell migration was detected with the Transwell Migration assay. *P < 0.05, **P < 0.01 *vs.* respective controls.

We first confirmed that linc00152 was mainly located in the cytoplasm of HUVECs by RNA-FISH (Figure 3C), which is an important premise for a ceRNA. Then, the luciferase reporter gene assay was used to validate the binding of miR-4767 with linc00152. MiR-4767 suppressed the luciferase activity of Luc-linc00152-WT, but had no obvious effect on Luc-linc00152-MUT (Figure 4D). MiRNAs usually function in the form of miRNA-ribonucleoprotein complexes (miRNPs, also known as the RNA-induced silencing complex (RISC)**,** of which Ago2 is the core component. We found that linc00152 was preferentially enriched in Ago2-containing miRNPs compared to immunoglobulin G (IgG) immunoprecipitates (Figure 4E). Additionally, we produced biotinylated miR-4767 probes, including a WT probe and a seed sequence mutated probe, to verify their direct binding. The results depicted that the WT biotinylated miR-4767 probe could pull down linc00152, but the MUT biotinylated miR-4767 probe could not (Figure 3F). Moreover, after overexpression or knockdown of linc00152 for 48 h and oxLDL treatment for 24 h, the level of miR-4767 in HUVECs decreased and increased respectively (Figure 3G). Similarly, linc00152 expression was suppressed and increased respectively when the cells were transfected with miR-4767 mimic or antagomir (Figure 3H). Therefore, linc00152 could bind with miR-4767 as a miRNA sponge and it decreased the expression of miR-4767.

### MiR-4767 induced apoptosis and suppressed migration in HUVECs through targeting Bcl2L12 and EGFR

**Figure 5:**
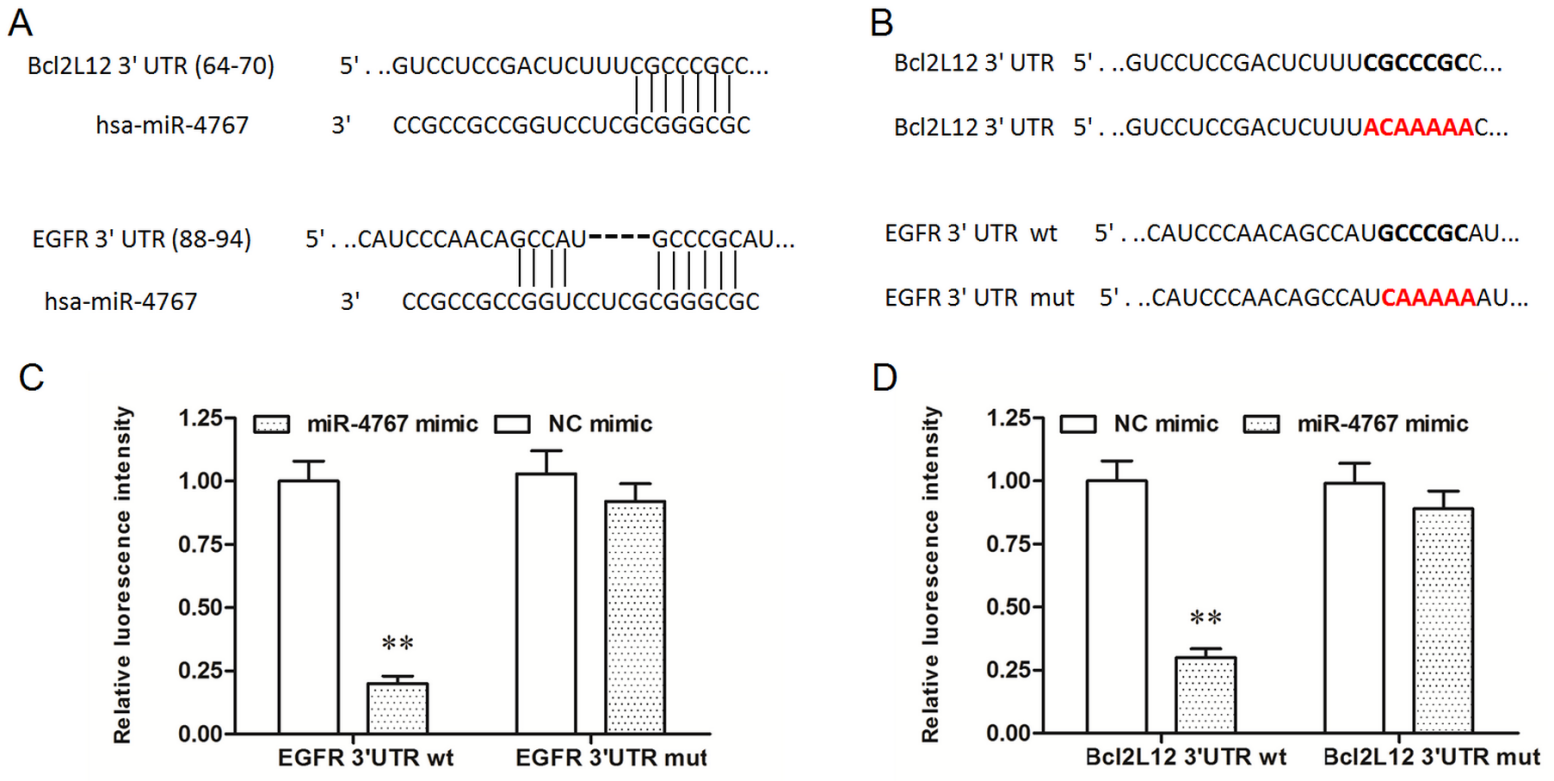
MiR-4767 directly targets Bcl2L12 and EGFR **(A)** Potential sited targeted by miR-4767 in the sequences of Bcl2L12 and EGFR 3’UTRs. **(B)** wt and mut sequences of Bcl2L12 or EGFR inserted into the luciferase reporter gene vectors. Luc-Bcl2L12 3’UTR-wt or Luc-Bcl2L12 3’UTR-mut vectors **(C)** and Luc-EGFR 3’UTR-wt or Luc-EGFR 3’UTR-mut vectors **(D)** were co-transfected into HEK293T cells with 40 nM miR-4767 mimics or NC mimics for 48 h and luciferase activity was measured. **P < 0.01 *vs.* NC mimics.

**Figure 6:**
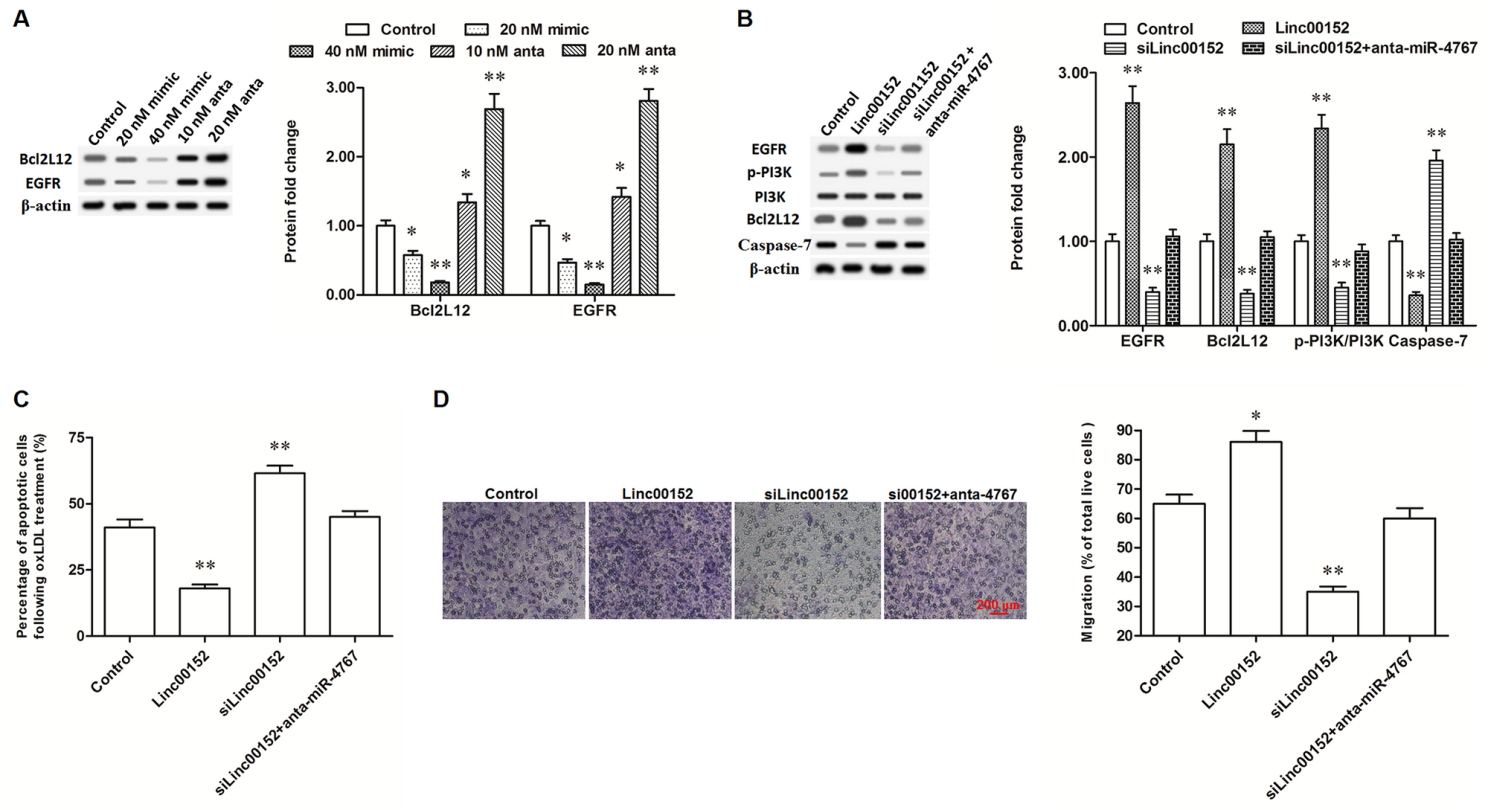
Linc00152 promoted the expression of Bcl2L12 and EGFR through diminishing miR-4767 **(A)** MiR-4767 negatively regulated the protein levels of Bcl2L12 and EGFR. HUVECs were transfected with miR-4767 mimics at concentrations of 20, and 40 nM or miR-4767 antagomirs at 10 and 20 nM for 48 h. Then, the cells were treated with 150 μg/mL ox-LDL for 24 h and the protein levels of Bcl2L12 and EGFR were detected by Western blotting. **(B)** Linc00152 promoted the expression of Bcl2L12 and EGFR through diminishing miR-4767. **(C and D)** Block of miR-4767 antagonized the changes of apoptosis and migration in HUVECs caused by linc00152 knockdown. HUVECs were transfected with 1.0 μg/mL pcDNA3.1-linc00152, 40 nM Linc00152 siRNA, or 40 nM Linc00152-siRNA plus 20 nM miR-4767 antagomirs. After incubation for 48 h, the cells were treated with 150 μg/mL ox-LDL for 24 h. The protein levels of Bcl2L12 and EGFR were detected by Western blotting. Cell apoptosis was checked by TUNEL assay and cell migration was detected with the Transwell Migration assay. *P < 0.05, **P < 0.01 *vs.* control.

It was recently shown that miR-4767 induced apoptosis in HUVECs by targeting Bcl2L12. Here we confirmed that miR-4767 could directly target Bcl2L12 by bioinformatic and luciferase reporter gene assays (Figure 4A–4C). However, the role of miR-4767 in migration of HUVECs has not been defined. We searched for the potential miR-4767 target genes which might be associated with VEC migration. The results from RNAhybrid showed that EGFR, an important receptor in VEGF/PI3K/Akt pathway, was a potential target of miR-4767 (Figure 4A). The EGFR 3’UTR luciferase reporter gene assay verified the prediction and showed that miR-4767 suppressed the luciferase activity of Luc-EGFR-WT, but had no obvious effect on Luc-EGFR-MUT (Figure 5B, 5C and 5D). Furthermore, the miR-4767 mimic suppressed the expression of Bcl2L12 and EGFR proteins in a dose-dependent manner, while the miR-4767 antagomir upregulated those in a dose-dependent manner (Figure 6A). These data demonstrated that miR-4767 suppressed VEC function through inducing cell apoptosis and suppressing cell migration.

### Blocking miR-4767 rescued the decrease of Bcl2L12 and EGFR caused by linc00152 knockdown, reduced changes in apoptosis and migration in HUVECs

CeRNAs regulate other RNA transcripts, usually mRNAs, by competing for shared miRNAs. Finally, we verified the effect of linc00152 on Bcl2L12 and EGFR. According to our data, overexpression of linc00152 improved the expression of Bcl2L12, while knockdown of linc00152 suppressed the expression of Bcl2L12 (Figure 6B). As a result, the level of cleaved caspase-7, which could be neutralized by Bcl2L12, was decreased by linc00152 overexpression and increased by linc00152 knockdown (Figure 6B). Meanwhile, EGFR expression was also increased by linc00152 overexpression and suppressed by linc00152 knockdown (Figure 6B). Resultantly also, PI3K, an important downstream kinase of EGFR, was activated by linc00152 overexpression and inactivated by linc00152 knockdown (Figure 6B). Linc00152 siRNA transfection did not change the expression or activation of Bcl2L12, EGFR or their downstream proteins, when miR-4767 was blocked by the miR-4767 antagomir in HUVECs (Figure 6B). These data indicated that inhibition of miR-4767 rescued the decrease of Bcl2L12 and EGFR caused by linc00152 knockdown. Consistent with the change in protein levels, block of miR-4767 reduced the changes of apoptosis and migration in HUVECs caused by linc00152 knockdown (Figure 6C and 6D).

## DISCUSSION

Linc00152 was primitively discovered in an analysis for lncRNA expression profiles in gastric cancer [24]. This 828-bp lncRNA is located in the intergenic region between the pseudogenes Platelet Activating Factor Acetylhydrolase 1b Regulatory Subunit 1 Pseudogene 1 (PAFAH1B1P1) and LOC107985796 (Supplementary Figure 3). During the past 3 years, linc00152 has been shown to upregulated in several carcinoma tissues, promote cell proliferation and metastasis, and inhibit cell apoptosis [25–28]. However, its role in non-cancer biological processes was unknown. Recent studies revealed that linc00152 could be expressed in the circulatory system and that it responds to chemical stresses, suggesting that its function might be not restricted in carcinogenesis [29–31]. In this study, we found that linc00152 was downregulated in HUVECs treated with oxLDL, which is a typical proinflammatory factor in the initiation and progression of vascular endothelial dysfunction, in a dose- and time-dependent manner. Then, gain- and loss-function experiments indicated that linc00152 distinctly inhibited apoptosis and improved migration in oxLDL-treated HUVECs. Moreover, we revealed that linc00152 positively regulated Bcl2L12 and EGFR to exert its function in HUVEC apoptosis and migration, by sponging their shared miRNA--miR-4767.

The molecular mechanisms of lncRNAs are quite diverse. For example, up to date, linc00152 has displayed at least three mechanisms in different cell types. In hepatocellular carcinoma cells, linc00152 functions as a “transcription factor”. It directly bound to the promoter of the epithelial cell adhesion molecule and then activated the mechanistic target of the rapamycin (mTOR) pathway [26]. In gastric cancer cells, linc00152 can function as either a “signal transductor” or a “scaffold”. RNA pull-down and RNA-immunoprecipitation assays showed that linc00152 could directly bind with the EGFR and activate PI3K/AKT signaling [27]; it has also been shown that linc00152 can bind to the enhancer of zeste homolog 2 (EZH2), which is the core of the polycomb group, and then epigenetically regulates multiple genes involved in cell cycle control [25]. In this study, we found that linc00152 functioned as a ceRNA in the suppression of Bcl2L12 mediated apoptosis and the improvement of EGFR induced migration in HUVECs by sponging miR-4767. Similarly, a recent study on the lncRNA microarray in gastric cancer has revealed that linc00152 and other six other lncRNAs form a cancer-associated ceRNA network with 9 miRNAs, including miR-18a-5p, miR-18b-5p, miR-19a-3p, miR-20b-5p, miR-106a-5p, miR-106b-5p, miR-31-5p, miR-139-5p, and miR-195-5p [32]. Moreover, a study on oxaliplatin resistance regulation in colon cancer also revealed that linc00152 is involved in the linc00152/miR-193a-3p/erb-b2 receptor tyrosine kinase 4 (ERBB4) ceRNA signaling axis [33]. Our findings propose two novel linc00152-participating ceRNA axes: linc00152/miR-4767/Bcl2L12 and linc00152/miR-4767/EGFR.

MiR-4767 was discovered in a comprehensive expression analysis of breast cancer [34]. A quite recent study revealed that miR-4767 was involved in HUVEC apoptosis by targeting the anta-apoptotic gene Bcl2L12 [22]. It is widely recognized that a miRNA can target different genes. In this study, we searched the potential target genes of miR-4767 and confirmed Bcl2L12 and EGFR are both target genes of miR-4767. Like Bcl2L12, which is a typical anta-apoptotic gene, EGFR is known for its positive role in cell survival and migration through activating multiple kinase pathways, such as PI3K/AKT. By targeting EGFR, miR-4767 could suppress HUVEC migration.

In summary, linc00152 sponges miR-4767 to abate its suppression of Bcl2L12 and EGFR, suppressing apoptosis and promoting migration in vascular endothelial cells. Our findings propose a novel role of linc00152 in the regulation of vascular endothelial function.

## MATERIALS AND METHODS

### Cell culture and ox-LDL treatment

Human umbilical vein endothelial cells (HUVECs) were purchased from American Type Culture Collection (ATCC) Inc. (Manassas, VA, USA). HEK293T cells were obtained from the Cell Resource Center of Shanghai Institute of Life Science (Shanghai, China). The cells were cultured in Dulbecco’s Modified Eagle Medium (DMEM, Invitrogen, Carlsbad, CA, USA) supplemented with 10% fetal bovine serum (FBS, Invitrogen), 100 U/mL penicillin (Invitrogen) and 100 mg/mL streptomycin (Invitrogen). The cells were incubated at 37°C in a humidified and 5%-CO_2_ atmosphere.

For treatment, HUVECs were subcultured in 12-well culture plates at a density of 10^5^/well. On reaching 50% confluence, ox-LDL (dissolved in PBS containing 0.08 μM EDTA-Na_2_) was added into the cells at the final concentration of 50, 100, 150 and 200 μg/mL. As a control, an equal volume of PBS containing 0.08 μM EDTA-Na_2_ was incubated with the cells.

### Transfection of vectors, siRNAs and miRNA mimics/antagomirs

The pcDNA3.1 (+) vector containing the full-length linc00152 sequence and empty vector were purchased from GenePharma (Shanghai, China). The linc00152 siRNAs (siLinc00152), miR-4767 mimics and miR-4767 antagomirs (anta-miR-4767), as well as scrambled negative control oligos, were synthesized by Ribobio Inc. (Guangzhou, China). The cells at 70% confluence were transfected with constructs or oligos using Lipofectamine 3000 (Invitrogen) according to the manufacturer’s instructions.

### Real-time quantitative PCR (qPCR)

Total RNA of the cells was extracted with the Trizol agent (Invitrogen). The cDNA was synthesized with the Superscript III Reverse Transcriptase Kit (Invitrogen) according to the manufacturer’s instructions. The qPCR reactions were carried out in a final volume of 25 μL with a SYBR Premix Ex Taq II Kit (TaKaRa, Dalian, China), run in triplicate wells, and analyzed by the iQ™5 Multicolor Real-Time PCR Detection System (Bio-Rad, Hercules, CA, USA). The reaction profile was as follows: 95°C for 1 min followed by 35 cycles of 94°C for 15 s, 54°C for 30s, and 72°C for 20s. The transcript abundances of all tested genes were calculated using the 2^-ΔΔCt^ method and normalized to U6 RNA. The primers used in the reactions were designed and synthesized by Ribobio Inc.

### Western blotting

Thirty μg of protein was separated by 12% SDS-PAGE and transferred onto a 0.22-μm PVDF membrane (Millipore, Boston, MA, USA). The following primary antibodies were used to incubated the membrane at 4°C overnight: anti-Bcl2L12 (1:500, Abcam, Cambridge, UK), anti-EGFR (1:500, Abcam), anti-PI3K (1:400, Cell Signaling Technology (CST), Boston, MA, USA), anti-p-PI3K (1:200, CST), anti-caspase-7 (1:300, Abcam), and anti-β-actin (1:800, Abcam) that was used as the internal reference. After washing and incubating with the corresponding HRP-conjugate secondary antibodies, the membrane was analyzed with the Enhanced Chemiluminescent (ECL) Western blotting Kit (Millipore) in a Gel Imaging System (Bio-Rad).

### RNA fluorescent in situ hybridization (RNA-FISH)

Linc00152 subcellular localization was detected with a FISH Kit (Roche, Basel, Switzerland). HUVECs were collected, washed twice and fixed with 4% paraformaldehyde. Then, hybridization solution was added into the plates, which were incubated with a digoxigenin-labeled linc00152 probe (Sigma, St. Louis, MO, USA). A scrambled probe was used as a negative control. The cell nucleus was stained with DAPI (Sigma) for 10 min at room temperature. After wishing twice with ice-cold PBS, fluorescence images were captured by a Confocal Laser Scanning Microscope (FV1000, Olympus, Tokyo, Japan).

### Detection of cell apoptosis and migration

Cell apoptosis was detected with a Terminal deoxynucleotidyl transferase-mediated dUTP nick end labeling (TUNEL) kit (Roche, Basel, Switzerland) following the manufacturer’s instructions. The apoptotic ratio was quantified by calculating the number of positive cells from a total of 1000 cells in 12 random fields.

### Luciferase constructs and activity detection

Wild type (WT) BclL12 3’UTR, EGFR 3’UTR and linc00152 sequences were individually amplified by PCR. The mutations of the sequences (MUT) were generated using the QuikChange II XL Site-Directed Mutagenesis Kit (Stratagene, Palo Alto, CA, USA). The WT sequences and MUT sequences devoid of the miR-4767 binding site were respectively subcloned into the Dual-Glo Dual-Luciferase vector (Promega, Madison, WI, USA) downstream from the coding region of the luciferase gene. HEK293T cells were seeded into 24-well plates at 3 × 10^4^ cells per well. On reaching 70% confluence, the cells were co-transfected with constructs of dual-luciferase (firefly and Renilla luciferase) reporters and miRNA mimics or NC oligos with Lipofectamine 3000 for 48 h. Dual luciferase activity was measured according to the instructions of the Dual-Glo Dual-Luciferase kit. Firefly luciferase or Renilla luciferase activity was measured by Multilabel Plate Reader (VICTOR X2, PerkinElmer, USA). Firefly luciferase activity was normalized to that of Renilla.

### Pull-down assay with biotinylated miRNA

The HUVECs were transfected with WT biotinylated miR-4767 or the MUT biotinylated miR-4767 (50 nM). After 48 h, the cells werecollected andwashed with PBS followed by vortex. Then, the cells were incubated in a specific lysis buffer (Ambion, Austin, Texas, USA) for 10 min. 50 mL of lysates from the sample was aliquoted for input. The residual lysates were incubated with M-280 streptavidin magnetic beads(Sigma) that was pre-coated with RNase-free BSA and yeast tRNA (Sigma). The beadswere incubated at 4°C for 3 h, washed twice with ice-cold lysis buffer, three timeswith the low salt buffer and once with the high salt buffer. The bound RNAs were purified by Trizol and then applied in the qPCR analysis for lncRNA-Linc00152 enrichment.

### Statistical analysis

All data were obtained from at least three independent experiments. Values were expressed as means ± SEM. Statistics were calculated with SPSS statistics v23.0 software. Multiple comparisons were assessed by one-way ANOVA followed by Dunnett’s tests. The difference between groups was considered statistically significant if *P* < 0.05.

## SUPPLEMENTARY MATERIALS FIGURES


