# Supplementary material for: Linc00152 suppresses apoptosis and promotes migration by sponging miR-4767 in vascular endothelial cells
Source: Oncotarget. 2017 Jun 28;8(49):85014–23. doi: 10.18632/oncotarget.18777 (PMC5689590; doi:10.18632/oncotarget.18777)
Supplement: Supplementary file 1 [file oncotarget-08-85014-s001.pdf]

Linc00152 suppresses apoptosis and promotes migration by sponging miR-4767 in vascular endothelial cells

SUPPLEMENTARY MATERIALS

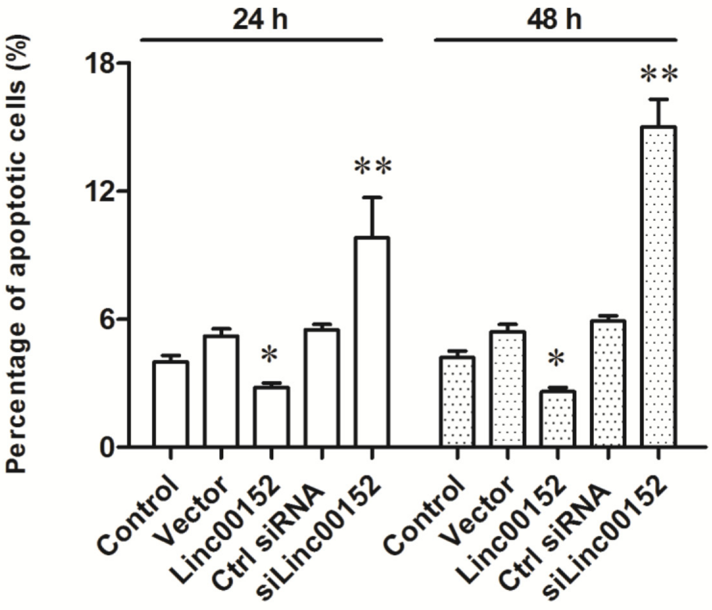

Supplementary Figure 1: Linc00152 suppressed apoptosis in HUVECs under normal condition. HUVECs were transfected with 1.0 µg/mL pcDNA3.1-linc00152 or 40 nM Linc00152 siRNA. At 24 h and 48 h, cell apoptosis was checked by TUNEL assay.

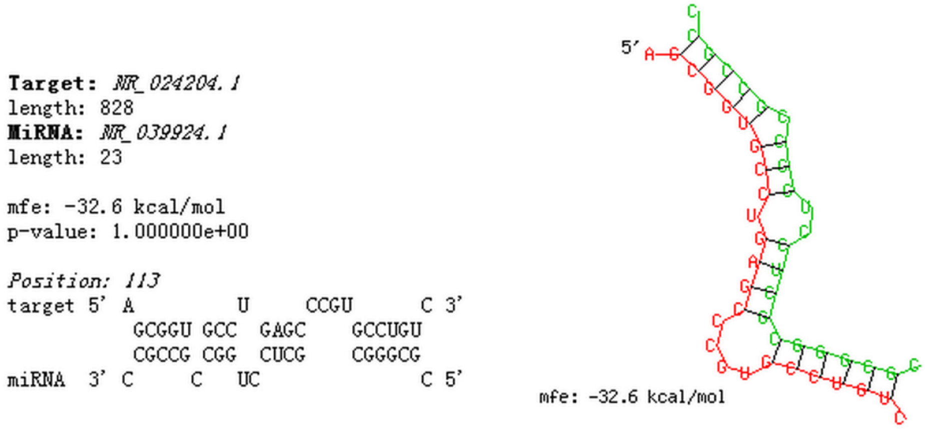

Supplementary Figure 2: The binding details of linc00152 and miR-4767 predicted by RNAHybrid. Mfe: molecular binding free energy.

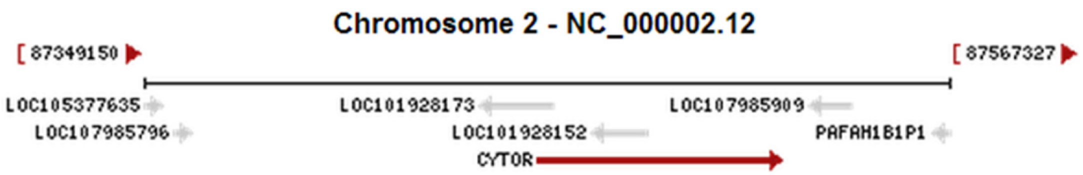

Supplementary Figure 3: The location of linc00152 gene (also CYTOR) on Chromosome2.
